# Joint Analysis of Multiple Phenotypes in Association Studies based on Cross-Validation Prediction Error

**DOI:** 10.1038/s41598-018-37538-y

**Published:** 2019-01-31

**Authors:** Xinlan Yang, Shuanglin Zhang, Qiuying Sha

**Affiliations:** Department of Mathematical Sciences, Michigan Technological University, Houghton, Michigan United States of America

## Abstract

In genome-wide association studies (GWAS), joint analysis of multiple phenotypes could have increased statistical power over analyzing each phenotype individually to identify genetic variants that are associated with complex diseases. With this motivation, several statistical methods that jointly analyze multiple phenotypes have been developed, such as O’Brien’s method, Trait-based Association Test that uses Extended Simes procedure (TATES), multivariate analysis of variance (MANOVA), and joint model of multiple phenotypes (MultiPhen). However, the performance of these methods under a wide range of scenarios is not consistent: one test may be powerful in some situations, but not in the others. Thus, one challenge in joint analysis of multiple phenotypes is to construct a test that could maintain good performance across different scenarios. In this article, we develop a novel statistical method to test associations between a genetic variant and Multiple Phenotypes based on cross-validation Prediction Error (MultP-PE). Extensive simulations are conducted to evaluate the type I error rates and to compare the power performance of MultP-PE with various existing methods. The simulation studies show that MultP-PE controls type I error rates very well and has consistently higher power than the tests we compared in all simulation scenarios. We conclude with the recommendation for the use of MultP-PE for its good performance in association studies with multiple phenotypes.

## Introduction

Traditionally, genome-wide association studies (GWAS) have performed on individual phenotype. In spite of the success of GWAS in identifying thousands of associations between genetic variants and complex diseases, these identified variants only contribute to a small proportion of the phenotypic variation. In the study of a complex disease, several correlated phenotypes are usually measured for a disorder or its risk factors^1^, therefore, by jointly analyzing multiple correlated phenotypes, we may increase statistical power to detect causal variants with weak genetic effects on complex diseases.

One method to use multiple phenotypes in association studies is to analyze each phenotype separately as the standard univariate association test and then aggregate the results. This approach will have a loss in power due to the penalties from the multiple testing^1,2^ and the ignorance of the correlation structure among phenotypes^3,4^. Thus, multiple-phenotype association study that uses multiple phenotypes simultaneously has become popular.

Several methods to detect association using multiple phenotypes simultaneously have been introduced in recent years. For example, O’Brien method (OB) is proposed to combines test statistics obtained from association test for each individual phenotype^5^. OB is the most powerful test when the genetic effects are homogeneous and loses power when genetic effects are heterogeneous, especially when genetic effects have opposite directions^1,6^. van der Sluis *et al*.^7^ proposed a trait-based association test using an extended Simes procedure (TATES) that conducts association test for each phenotype and then combines the univariate p-values while correcting for the correlation between p-values. The canonical correlation analysis (CCA) conducts the linear combination of phenotypes that explain the largest amount of correlation between a genetic variant and phenotypes^8^. One could also use multivariate analysis of variance (MANOVA) in regression to study multiple phenotypes^9^. MANOVA is equivalent to CCA when canonical correlation analysis is applied to a single variant^10^. MultiPhen proposed by O’Reilly *et al*.^2^ can be used to detect the association between one variant and multiple phenotypes by reversing response and predictors via a proportional odds regression model. When a small number of phenotypes are included, MultiPhen and MANOVA lead to similar performance^6,11^. MANOVA and CCA require the assumption of normality of multiple phenotypes, while MultiPhen has no inflated type I error rates on non-normal phenotypes^2^. Some other variable reduction methods have also been proposed to test for the association between a genetic variant and the linear combination of multiple phenotypes rather than the original phenotypes^12–14^. For example, principal component of phenotypes (PCP) that maximizes the phenotype variation is the most popular dimension reduction method^13^. Based on PCP, Klei *et al*.^12^ developed principal component of heritability (PCH) by maximizing the heritability among all linear combination of phenotypes. Recently, Turley *et al*.^15^ introduced the Multi-Trait Analysis of GWAS (MTAG) for joint analysis of multiple phenotypes. MTAG can be applied to GWAS summary statistics from an arbitrary number of phenotypes without access to individual-level data.

Although there are many proposed methods for joint analysis of multiple phenotypes, the performance of these methods under a wide range of scenarios is not consistent^6^: one test may be powerful in some situations, but not in the others. Thus, one challenge in multiple phenotype analysis is to construct a test that could maintain good performance across different scenarios. In this article, we develop a novel statistical method to test the association between a genetic variant and Multiple Phenotypes based on cross-validation Prediction Error (MultP-PE). Extensive simulation studies are conducted to evaluate the type I error rates and to compare the power performance of MultP-PE with various existing methods. Our simulation studies show that MultP-PE controls the type I error rates very well and has consistently higher power than other methods we compared in all simulation scenarios.

## Method

We consider a sample with *n* unrelated individuals. Each individual has *K* (potentially correlated) phenotypes and has been genotyped at a variant of interest. Let *y*_*ik*_ denote the *k*^*th*^ phenotype value of the *i*^*th*^ individual and *x*_*i*_ denote the genotype score of the *i*^*th*^ individual, where *x*_*i*_∈{0, 1, 2} is the number of minor alleles that the *i*^*th*^ individual carries. We model the relationship between the multiple phenotypes and the genetic variant using an inverse linear regression model, in which the genotype at the variant of interest is the response variable and the multiple phenotypes are predictors. That is,

1 $${x}_{i}={\beta }_{0}+{\beta }_{1}{y}_{i1}+\ldots +{\beta }_{K}{y}_{iK}+{\varepsilon }_{i}.$$

We are not the first using an ordinal variable as response variable in a linear model. To correct for population stratification, Price *et al*.^16^ used a qualitative phenotype or genotypes as response variables in linear models. To adjust the effects of covariates in rare variant association studies, Sha *et al*.^17^ also used a qualitative phenotype or genotypes as response variables in linear models. To test the association between the *K* multiple phenotypes and the variant, we test the null hypothesis *H*_0_:*β*_1_ = ··· = *β*_*K*_ = 0 under model (1).

Let *y*_*i*_ = (1,*y*_*i*1_, …, *y*_*iK*_)^*T*^ and *β* = (*β*_0_, *β*_1_, …, *β*_*K*_)^*T*^, then the regression model in equation (1) can be written as ${x}_{i}={y}_{i}^{T}\beta +{\varepsilon }_{i},i=1,2,\ldots ,n$. The ordinary linear square estimate of *β* is $\hat{\beta }={({Y}^{T}Y)}^{-1}{Y}^{T}x$, where *Y* = (*y*_1_, …, *y*_*n*_)^*T*^ and *x* = (*x*_1_, …, *x*_*n*_)^*T*^. When multiple phenotypes are highly correlated, the rank of matrix *Y* may be less than *K*, then the inverse of *Y*^*T*^*Y* may not exist, which results in that the ordinary linear square estimate of *β* may not be unique^18^. Since multiple phenotypes in a GWAS are usually highly correlated, we propose to use Ridge regression^19–24^. Ridge regression penalizes the size of the regression coefficients. The Ridge regression estimator of *β* is defined as the value of *β* that minimizes

$$\sum _{i}{({x}_{i}-{y}_{i}^{T}\beta )}^{2}+\lambda \sum _{j}{\beta }_{j}^{2},$$

where *λ* (*λ* ≥ 0) is a tuning parameter. The solution to the Ridge regression is given by ${\hat{\beta }}_{\lambda }={({Y}^{T}Y+\lambda I)}^{-1}{Y}^{T}x$. Here the estimator of *β* depends on *λ* and we use the subscript *λ* to indicate that the estimator of *β* is a function of *λ*.

Based on Ridge regression, we propose to use the leave-one-out cross validation (LOOCV) prediction error under model (1) as a test statistic. Let ${\hat{x}}_{-i}^{\lambda }$ denote the LOOCV predicted value (leave the *i*^*th*^ individual out) of *x*_*i*_ under model (1) with parameter *λ* in Ridge regression. Then, the statistic can be written as ${T}_{\lambda }=\sum _{i=1}^{n}{({x}_{i}-{\hat{x}}_{-i}^{\lambda })}^{2}$. Note that *T*_*λ*_ is the LOOCV prediction error, thus low values of *T*_*λ*_ would imply significance. Let *p*_*λ*_ denote the p-value of *T*_*λ*_ (see next paragraph on how to calculate *p*_*λ*_). We define the test statistic of Multiple Phenotypes based on Prediction Error (MultP-PE) as

2 $${T}_{MultP-PE}={\min }_{\lambda }{p}_{\lambda }.$$

We propose to use a grid search method in equation (2) to evaluate the minimization. We divide the interval [0, ∞) into subintervals $0\le {\lambda }_{1} < \cdot \cdot \cdot  < {\lambda }_{M-1} < {\lambda }_{M} < \infty$. Then, ${T}_{MultP-PE}={{\rm{\min }}}_{\lambda }{p}_{\lambda }={\min }_{1\le m\le M}{p}_{{\lambda }_{m}}$. We use a permutation procedure to evaluate the p-value of *T*_*MultP*−*PE*_. Intuitively, we need to use two layers of permutations to estimate ${p}_{{\lambda }_{m}}$ and the overall p-value for the test statistic *T*_*MultP*−*PE*_. For microarray data analysis, Ge *et al*.^25^ proposed that one layer of permutation can be used to estimate p-values. We use the permutation procedure of Ge *et al*. to estimate ${p}_{{\lambda }_{m}}$ and the overall p-value for the test statistic *T*_*MultP*−*PE*_. In each permutation, we randomly shuffle the genotypes at the variant. Suppose that we perform *B* times of permutations. Let ${T}_{{\lambda }_{m}}^{(b)}$ denote the value of ${T}_{{\lambda }_{m}}$ based on the *b*^*th*^ permuted data for *b* = 0, 1, …, *B* and *m* = 1, …, *M*, and ${p}_{{\lambda }_{m}}^{(b)}$ denote the p-value of ${T}_{{\lambda }_{m}}^{(b)}$, where *b* = 0 represents the original data. Then, we can estimate ${p}_{{\lambda }_{m}}^{(b)}$ using ${p}_{{\lambda }_{m}}^{(b)}=\frac{\#\{d:{T}_{{\lambda }_{m}}^{(d)} < {T}_{{\lambda }_{m}}^{(b)}\,{\rm{for}}\,d=1,\ldots ,B\}}{B}$. Let ${T}_{MultP-PE}^{(b)}={\min }_{1\le m\le M}{p}_{{\lambda }_{m}}^{(b)}$ denote the test statistic of *T*_*MultP*−*PE*_ based on the *b*^*th*^ permuted data, then the p-value of *T*_*MultP*−*PE*_ is given by

3 $$\frac{\#\{{T}_{MultP-PE\,}^{(b)}:{T}_{MultP-PE}^{(b)} < {T}_{MultP-PE}^{(0)}\,{\rm{for}}\,b=1,2,\ldots ,B\}}{B}$$

To apply MultP-PE to GWAS with hundreds of thousands of SNPs, we also propose an algorithm that can perform the permutation procedure described above more efficiently in the following section.

### A Fast Algorithm for the Permutation Procedure

We use the notations in the above section and let *A*_*λ*_ = (*Y*^*T*^*Y* + *λI*)^−1^, ${h}_{i}^{\lambda }={y}_{i}^{T}{A}_{\lambda }{y}_{i}$, $\,{h}_{\lambda }=({h}_{1}^{\lambda },\ldots ,{h}_{n}^{\lambda })$, and ${\hat{\beta }}_{\lambda }={A}_{\lambda }{Y}^{T}x$. Then, the Ridge predicted value of *x*_*i*_ is ${\hat{x}}_{i}^{\lambda }={y}_{i}^{T}{\hat{\beta }}_{\lambda }$ and ${\hat{x}}_{\lambda }={({\hat{x}}_{1}^{\lambda },\ldots ,{\hat{x}}_{n}^{\lambda })}^{T}=Y{({Y}^{T}Y+\lambda I)}^{-1}{Y}^{T}x$. We can show that the LOOCV prediction error in Ridge regression has a closed-form formula^24,26^, that is, ${x}_{i}-{\hat{x}}_{-i}^{\lambda }=({x}_{i}-{\hat{x}}_{i}^{\lambda })/(1-{h}_{i}^{\lambda })$. Note that for two matrices or vectors *A* and *B*, we use A*B and $\frac{A}{B}$ to denote the element-wise operations; for a matrix *C*, we use *colSum*(*C*) to denote the sums of the columns of matrix *C*. We assume *n* ≥ *K* + 1. We perform singular value decomposition of *Y*, that is, *Y* = *UDV*, where *U* is an *n* × (*K* + 1) matrix with orthonormal columns, *D* is (*K* + 1) × (*K* + 1) diagonal matrix with non-negative real numbers on the diagonal, and *V* is an (*K* + 1) × (*K* + 1) orthogonal matrix. Let *D* = *diag*(*d*_1_, …, *d*_*K* + 1_). Then, ${\hat{x}}_{\lambda }=U{C}_{\lambda }{U}^{T}x$, where *C*_*λ*_ = *diag*(*c*_*λ*,1_, …, *c*_*λ*,*K* + 1_) and ${c}_{\lambda ,j}={d}_{j}^{2}/({d}_{j}^{2}+\lambda )$ for *j* = 1, …, *K* + 1. Let ${c}_{\lambda }={({c}_{\lambda ,1},\ldots ,{c}_{\lambda ,K+1})}^{T}$ and *x*^(*K*)^ = *U*^*T*^*x* be a *K* + 1 dimensional vector. Then, ${\hat{x}}_{\lambda }=U{C}_{\lambda }{x}^{(K)}=U({c}_{\lambda }\ast {x}^{(K)})$ and *h*_*λ*_ = *diag*(*UC*_*λ*_*U*^*T*^). For $0\le {\lambda }_{1} < \ldots  < {\lambda }_{M} < \infty$, let $C=({c}_{{\lambda }_{1}},\ldots ,{c}_{{\lambda }_{M}})$ and $H=({h}_{{\lambda }_{1}},\ldots ,{h}_{{\lambda }_{M}})$. Then, $(\,{\hat{x}}_{{\lambda }_{1}},\ldots ,\,{\hat{x}}_{{\lambda }_{M}})=U(C\ast {x}^{(K)})=U({c}_{{\lambda }_{1}}\ast {x}^{(K)},\ldots ,{c}_{{\lambda }_{M}}\ast {x}^{(K)})$. If we denote $Q=\frac{(x-{\hat{x}}_{{\lambda }_{1}}\,,\ldots ,\,x-{\hat{x}}_{{\lambda }_{M}})}{1-H}$, then $({T}_{{\lambda }_{1}},\ldots ,{T}_{{\lambda }_{M}})=colSum(Q\ast Q)$. Note that *C*, *U*, and *H* only depend on phenotypes and *λ*_1_, …, *λ*_*M*_. Thus, *C*, *U*, and *H* do not change in each permutation. For a GWAS, *C*, *U*, and *H* also do not change at different SNPs. For 1,000 permutations on one SNP, our fast algorithm is about 20 times faster than the original algorithm (the original algorithm calculates *T*_*λ*_ by ${T}_{\lambda }=\sum _{i=1}^{n}{({x}_{i}-{\hat{x}}_{-i}^{\lambda })}^{2}$). To perform a GWAS with hundreds of thousands of SNPs, we can use the same approach as was suggested in Zhu *et al*.^14^, that is, we can first select SNPs that show evidence of association based on a small number of permutations (e.g. 1,000), then use a large number of permutations to test the selected SNPs. For example, in our real data analysis with 630,860 SNPs, we first performed 1,000 permutations and selected SNPs with *p*-*value* ≤ 0.005, then we performed 10^8^ permutations on the selected SNPs because SNPs with *p-value* > 0.005 are not significantly associated with phenotypes.

Although we use a permutation procedure to calculate the p-value of MultP-PE, by using our fast algorithm, we can use less than one day to perform a typical GWAS. In our read data analysis on COPD in the following section, we performed a GWAS with 5,430 individuals across 630,860 SNPs and seven phenotypes. We completed the analysis in 10 hours on Intel Xeon E5-2680v3 by using a single node.

In the above section, we describe MulP-PE without considering covariates. If covariates are needed to be considered, we can incorporate covariates using the following approach in MultP-PE. Suppose that there are total *G* covariates we would like to consider and let (*z*_*i*1_, …, *z*_*iG*_)^*T*^ denote the covariates for the *i*^*th*^ individual. We can adjust esch of the phenotypes by the covariates by applying the linear regression model ${y}_{ik}={a}_{0k}+{a}_{1k}{z}_{i1}+\ldots +{a}_{Mk}{z}_{iG}+{\varepsilon }_{ik}$, for *i* = 1, 2, …, *n*, *k* = 1, 2, …, *K*, and use the residual of *y*_*ik*_ to replace *y*_*ik*_ in MultP-PE. In our real data analysis, we used this approach to incorporate four covariates. This approach has been used in the literature. For example, Sha *et al*.^16^ and Zhu *et al*.^14^ also used the same approach to adjust phenotypes for the covariates.

In association studies for unrelated individuals, it has been well known that population stratification can seriously confound association results^27^. There are several methods that have been developed to control for population stratification. For example, Genomic Control (GC) approach^28,29^, Principal Component (PC) approach^16,30–32^, and Mixed Linear Model (MLM) approach^33,34^. Similar to most association tests for unrelated individuals, MulP-PE subjects to bias due to population stratification. To make MultP-PE robust to population stratification, we can use the PC approach. Let *c*_*i*1_, …, *c*_*iL*_ denote the top *L* PCs of the genotypes at a set of genomic markers for the *i*^*th*^ individual. We can use the residuals of the regression model ${x}_{i}=\alpha +{\beta }_{1}{c}_{i1}+\cdot \cdot \cdot +{\beta }_{L}{c}_{iL}+{\varepsilon }_{i}$ to replace *x*_*i*_ and use the residuals of the regression model ${y}_{ik}={\alpha }_{k}+{\beta }_{1k}{c}_{i1}+\cdot \cdot \cdot +{\beta }_{Lk}{c}_{iL}+{\varepsilon }_{ik}$ to replace *y*_*ik*_ for *k* = 1, 2, …, *K* in MultP-PE to adjust for population stratification.

### Comparison of Methods

We evaluate the performance of the proposed test MultP-PE by comparing it with five most commonly used methods for association studies using multiple phenotypes. These five methods include the O’Brien’s method (OB)^5^, Trait-based Association Test that uses Extended Simes procedure (TATES)^7^, Optimal weight method (OW)^6^, Multivariate analysis of variance (MANOVA)^9^, and Joint model of multiple phenotypes (MultiPhen)^2^.

## Simulation Study

In simulation studies, we evaluate type I error rates of MultP-PE by generating data sets with three different sample sizes, 500, 1,000 and 2,000. For power comparison, we compare the powers of different methods by simulation data sets with 1,000 unrelated individuals.

For genotype data, we generate genotype at a genetic variant according to minor allele frequency (MAF) and assume Hardy-Weinberg Equilibrium (HWE). For each individual, we generate *K* phenotypes using models similar to the models used in Zhu *et al*.^14^ and Wang *et al*.^35^. The *K* phenotypes are generated from the following model

4 $$y=\varphi x+c\gamma \omega +\sqrt{1-{c}^{2}}\times \varepsilon $$

where *y* = (*y*_1_, …, *y*_*K*_)^*T*^; *ϕ* = (*ϕ*_1_, …, *ϕ*_*K*_) are the genetic effects of the variant on the *K* phenotypes; *x* is the genotypic score at the variant; *c* is a constant number; *γ* is a *K* × *R* matrix; *ω* = (*ω*_1_, …, *ω*_*R*_)^*T*^ is a vector of factors with *R* elements and $\omega ={({\omega }_{1},\ldots ,{\omega }_{R})}^{T}\sim MVN(0,\Sigma )$, $\Sigma =\rho A+(1-\rho )I$, *ρ* is the correlation between factors, *A* is a matrix with elements of 1, and *I* is the identity matrix; *ε* = (*ε*_1_, …, *ε*_*K*_)^*T*^ is a vector of residuals, *ε*_1_, …, *ε*_*K*_ are independent, and ${\varepsilon }_{k}\sim N(0,1)$ for *k* = 1, …, *K*. Based on equation (4), we consider the following four models in which the within-factor correlation is *c*^2^ and the between-factor correlation is *ρc*^2^.

### Model 1

There is only one factor and genotypes impact on all phenotypes with different effect sizes. That is, *R* = 1, *ϕ* = *β*(1, 2, …, *K*)^*T*^, and *γ* = (1, …, 1)^*T*^.

### Model 2

There are two factors and genotypes impact on one factor. That is, *R* = 2, $\varphi ={(0,\ldots ,0,\mathop{\underbrace{\beta ,\ldots ,\beta }}\limits_{K/2})}^{T}$, and *γ* = *Bdiag*(*D*_1_, *D*_2_), where ${D}_{i}={(\mathop{\underbrace{1,\ldots ,1}}\limits_{K/2})}^{T}$ for *i* = 1, 2 and *Bdiag* means block diagonal.

### Model 3

There are five factors and genotypes impact on two factors. That is, *R* = 5, $\varphi ={({\beta }_{11},\ldots ,{\beta }_{1k},{\beta }_{21},\ldots ,{\beta }_{2k},{\beta }_{31},\ldots ,{\beta }_{3k},{\beta }_{41},\ldots ,{\beta }_{4k},{\beta }_{51},\ldots ,{\beta }_{5k})}^{T}$, and *γ* = *Bdiag*(*D*_1_, *D*_2_, *D*_3_, *D*_4_, *D*_5_), where ${D}_{i}={(\mathop{\underbrace{1,\ldots ,1}}\limits_{K/5})}^{T}$ for *i* = 1, …, 5; *k* = *K*/5; ${\beta }_{11}=\cdot \cdot \cdot ={\beta }_{1k}={\beta }_{21}=\cdot \cdot \cdot ={\beta }_{2k}={\beta }_{31}=\cdot \cdot \cdot ={\beta }_{3k}=0$; *β*_41_ = ··· = *β*_4*k*_ = −*β*; and $({\beta }_{51},\ldots ,{\beta }_{5k})=\frac{2\beta }{k+1}(1,\ldots ,k)$.

### Model 4

There are five factors and genotypes impact on four factors. That is, *R* = 5, $\varphi ={({\beta }_{11},\ldots ,{\beta }_{1k},{\beta }_{21},\ldots ,{\beta }_{2k},{\beta }_{31},\ldots ,{\beta }_{3k},{\beta }_{41},\ldots ,{\beta }_{4k},{\beta }_{51},\ldots ,{\beta }_{5k})}^{T}$, and *γ* = *Bdiag*(*D*_1_, *D*_2_, *D*_3_, *D*_4_, *D*_5_), where ${D}_{i}={(\mathop{\underbrace{1,\ldots ,1}}\limits_{K/5})}^{T}$ for *i* = 1, …, 5; *k* = *K*/5; *β*_11_ = ··· = *β*_1*k*_ = 0; *β*_21_ = ··· = *β*_2*k*_ = *β*; *β*_31_ = ··· = *β*_3*k*_ = −*β*; $({\beta }_{41},\ldots ,{\beta }_{4k})=-\,\frac{2\beta }{k+1}(1,\ldots ,k)$; and $({\beta }_{51},\ldots ,{\beta }_{5k})=\frac{2\beta }{k+1}(1,\ldots ,k)$.

For the type I error rates, we set *β* = 0 to indicate that the genetic variant has no effect on all phenotypes. For power comparisons, we consider different values of *β*. To evaluate type I error rate and power, we set MAF = 0.3, the between-factor correlation is 0.14, and the within-factor correlation is 0.25. In the following simulation studies and real data analysis, we use eight different values of *λ (M* = 8) and set $\mathrm{log}\,\lambda =0,\,1,\,2,\,3,\,3.5,\,3.8,\,4,\,4.5$.

The R codes for implementation of MultP-PE and for simulation of data under the four models are available at Dr. Shuanglin Zhang’s homepage http://www.math.mtu.edu/shuzhang/software.html.

## Results

To evaluate the type I error rates of MultP-PE, we consider different significance levels (0.01 and 0.05), different sample sizes (500, 1000 and 2000), and different number of phenotypes (10, 20 and 40). We use 1,000 permutations to calculate the p-values of MultP-PE and use 10,000 replicated samples to evaluate type I error rates of MultP-PE. For 10,000 replicated samples, the 95% confidence intervals (CIs) for the estimated type I error rates with nominal levels 0.05 and 0.01 are (0.04562, 0.05438) and (0.00804, 0.01196), respectively. We summarize the estimated type I error rates of the proposed test in Table 1. This table shows that only one type I error rate is not in the corresponding 95% CI (it is very close to the upper-bound of the CI), which indicates that the proposed method is valid.

**Table 1:** Estimated type I error rates for the MultP-PE method under four models.

Sample Size	Number of Phenotypes	Significance Level	Model 1	Model 2	Model 3	Model 4
500	10	*α* = 0.01	0.0103	0.0109	0.0112	0.0094
		*α* = 0.05	0.0480	0.0512	0.0523	0.0532
	20	*α* = 0.01	0.0116	0.0107	0.0114	0.0112
		*α* = 0.05	0.0503	0.0499	0.0473	0.0515
	40	*α* = 0.01	0.0112	0.0118	*0.0121*	0.0103
		*α* = 0.05	0.0524	0.0515	0.0518	0.0541
1000	10	*α* = 0.01	0.0108	0.099	0.0104	0.0116
		*α* = 0.05	0.0535	0.0532	0.0514	0.0492
	20	*α* = 0.01	0.0101	0.0095	0.0112	0.0083
		*α* = 0.05	0.0500	0.0501	0.0524	0.0469
	40	*α* = 0.01	0.0094	0.0116	0.0117	0.0105
		*α* = 0.05	0.0472	0.0512	0.0514	0.0508
2000	10	*α* = 0.01	0.0111	0.0094	0.0118	0.0094
		*α* = 0.05	0.0489	0.0491	0.0508	0.0465
	20	*α* = 0.01	0.0113	0.0107	0.0098	0.0108
		*α* = 0.05	0.0513	0.0491	0.0516	0.0523
	40	*α* = 0.01	0.0099	0.0091	0.0107	0.0110
		*α* = 0.05	0.0498	0.0480	0.0492	0.0476

The type I error rates are evaluated using 10,000 replicated samples. *P*-values of MultP-PE are estimated by 1,000 permutations. *α* is the significance level. The number of replications is 10,000. The type I error rate in italics indicates the value out of the bounds of the 95% CI.

**Figure 1:**
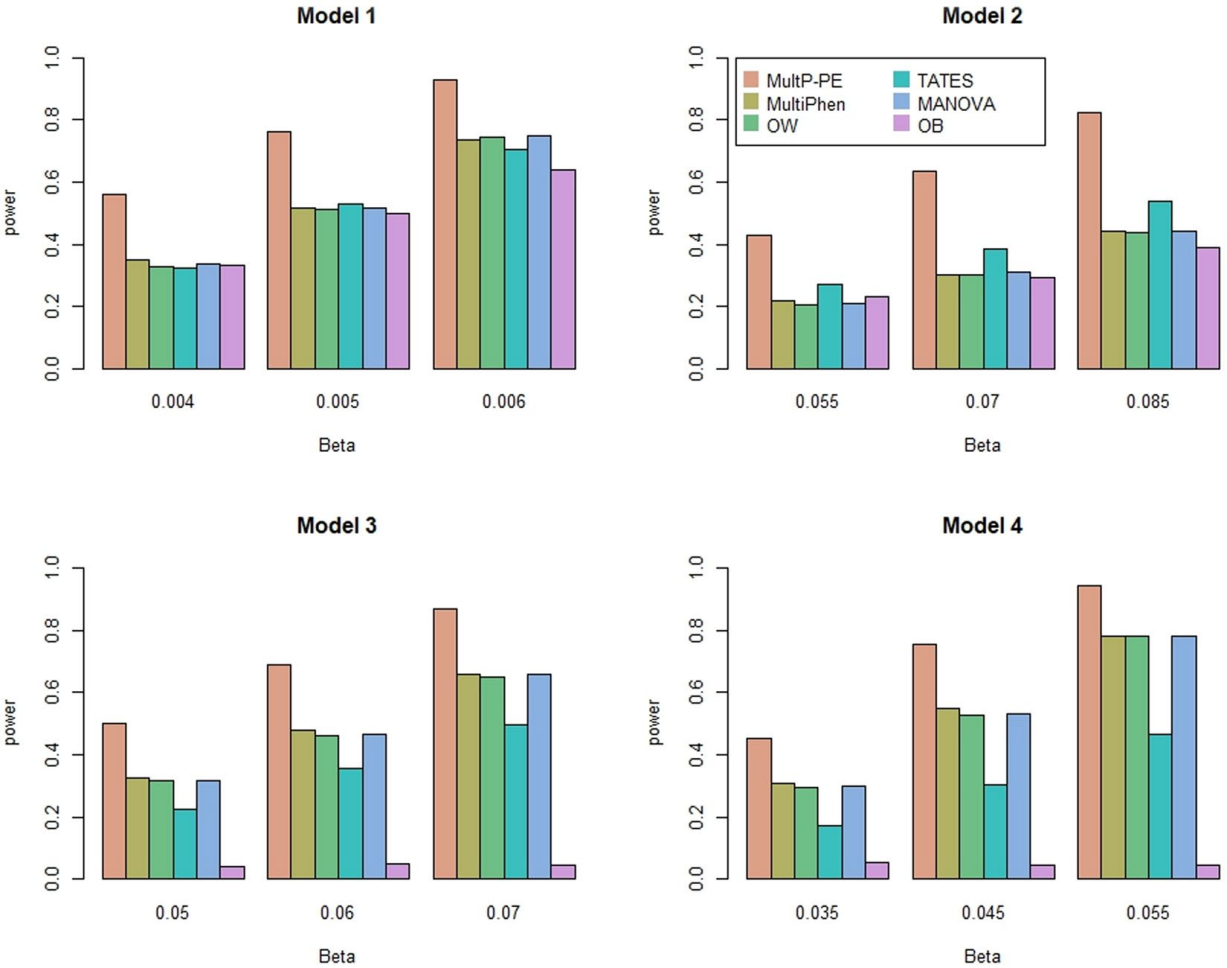
Power comparisons of the six methods as a function of effect size *β*. The total number of phenotypes is *K* = 20, sample size is 1000, MAF is 0.3, the between-factor correlation is 0.15, and the within-factor correlation is 0.25. Significance is assessed at the 5% level.

**Figure 2:**
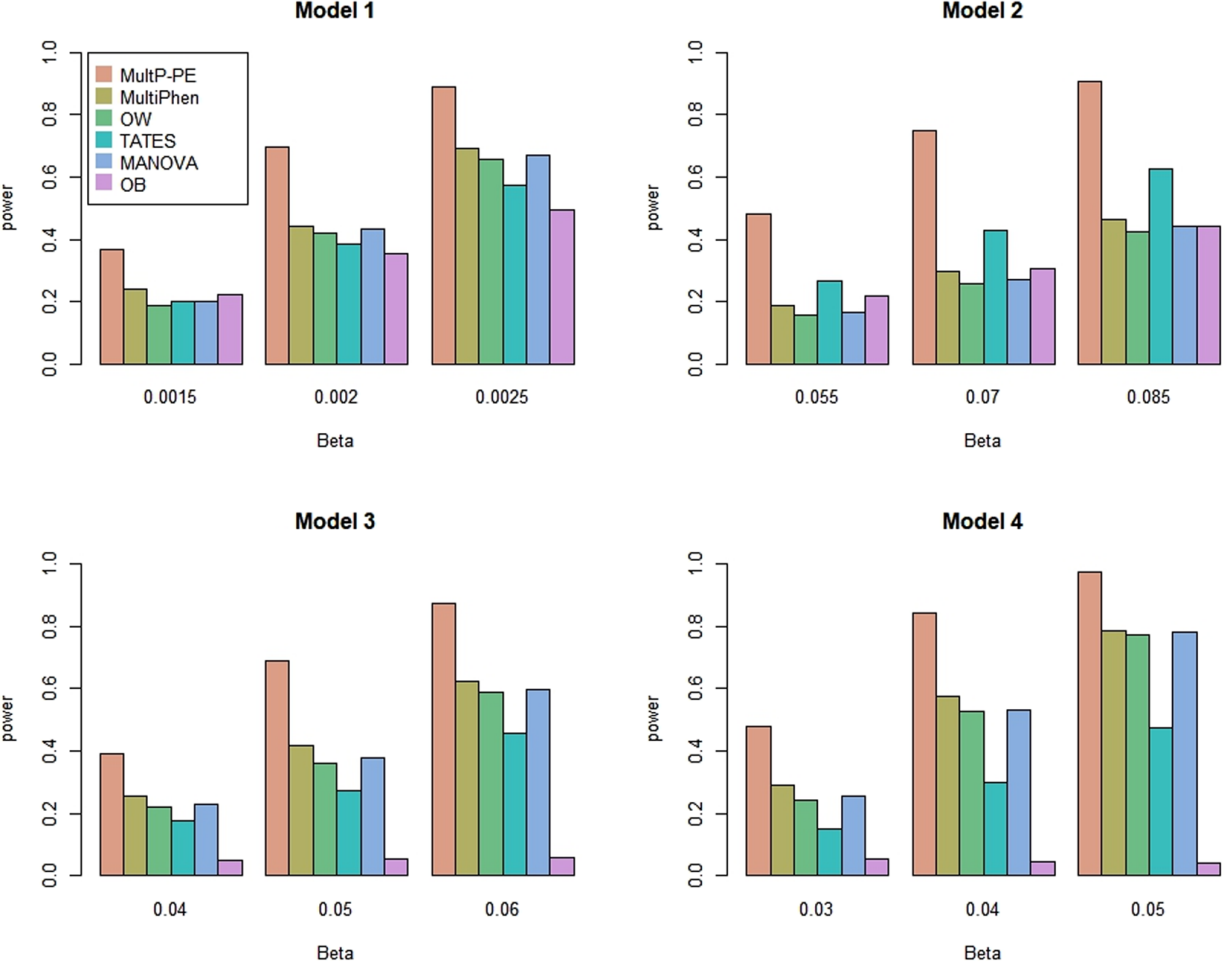
Power comparisons of the six methods as a function of effect size *β*. The total number of phenotypes is *K* = 40, sample size is 1000, MAF is 0.3, the between-factor correlation is 0.15, and the within-factor correlation is 0.25. Significance is assessed at the 5% level.

In power comparisons, we calculate the p-values of MultP-PE using 1,000 permutations and the p-values of MultiPhen, OW, TATES, MANOVA, OB using their asymptotic distributions. We evaluate the powers of all of the six tests using 1,000 replicated samples at a significance level of 0.05. Figures 1 and 2 show the powers of the six methods as a function of the effect size *β* with *K* = 20 and 40, respectively. As shown in these two figures: (1) MultP-PE is the most powerful test. The power of MultP-PE is much higher than the second most powerful test; (2) as the effect size *β* increases, the powers of all tests increase as well; as the number of phenotypes *K* increases from 20 to 40, MultP-PE presents more ascendancy than the other five tests; (3) MultiPhen, OW, and MANOVA have similar powers under all four models. A similar conclusion has been reached in some published papers^2,6,7^; (4) OB is comparable to MultiPhen, OW, and MANOVA in models 1 and 2, but has almost no power when the genetic effects have different directions (models 3 and 4); (5) TATES is more powerful than MultiPhen, OW, and MANOVA in model 2, but is less powerful than MultiPhen, OW, and MANOVA in models 3 and 4.

**Figure 3:**
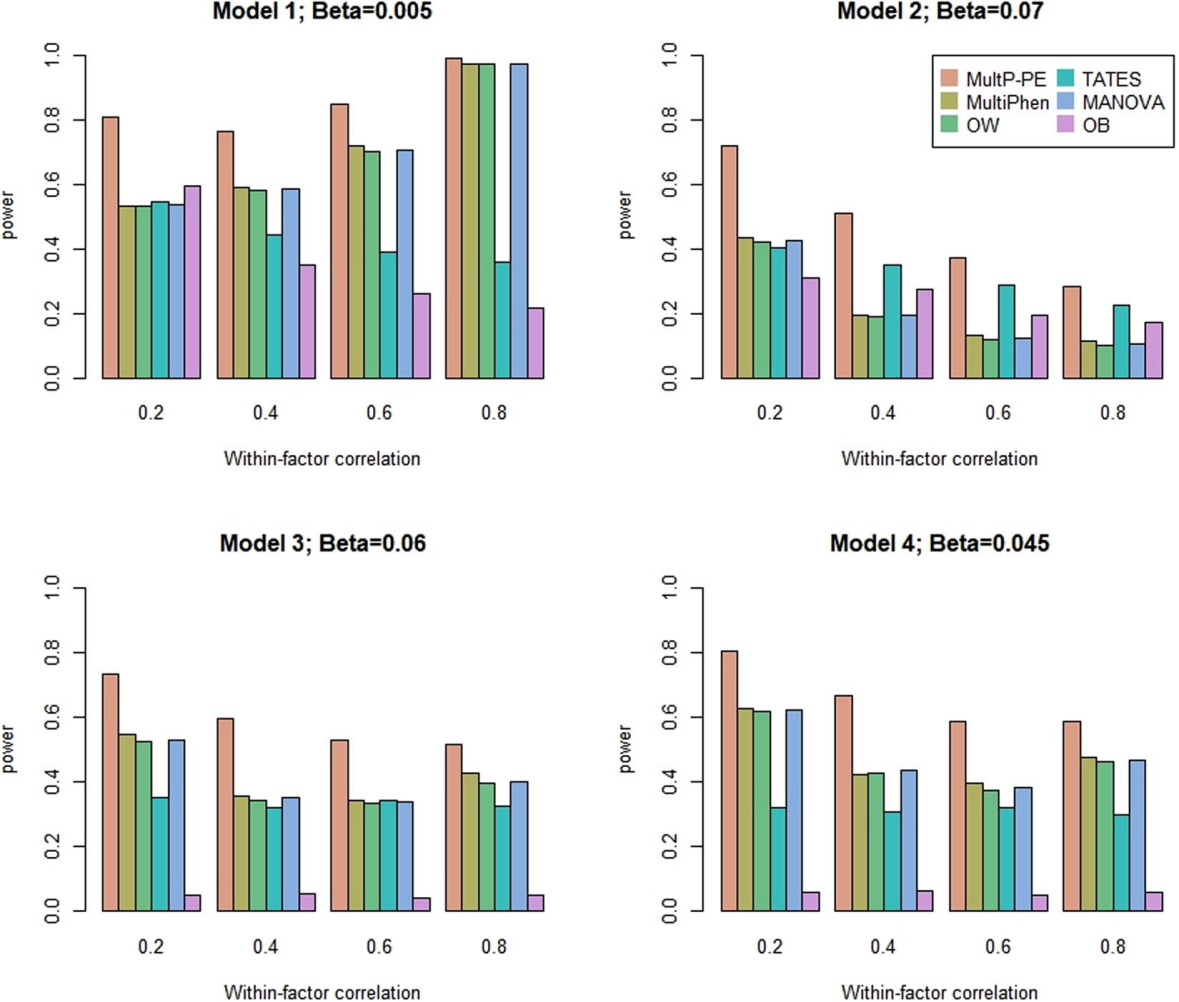
Power comparisons of the six methods as a function of within-factor correlation *c*^2^. The total number of phenotypes is *K* = 20, sample size is 1000, MAF is 0.3, and the between-factor correlation is 0.15. Significance is assessed at the 5% level.

**Figure 4:**
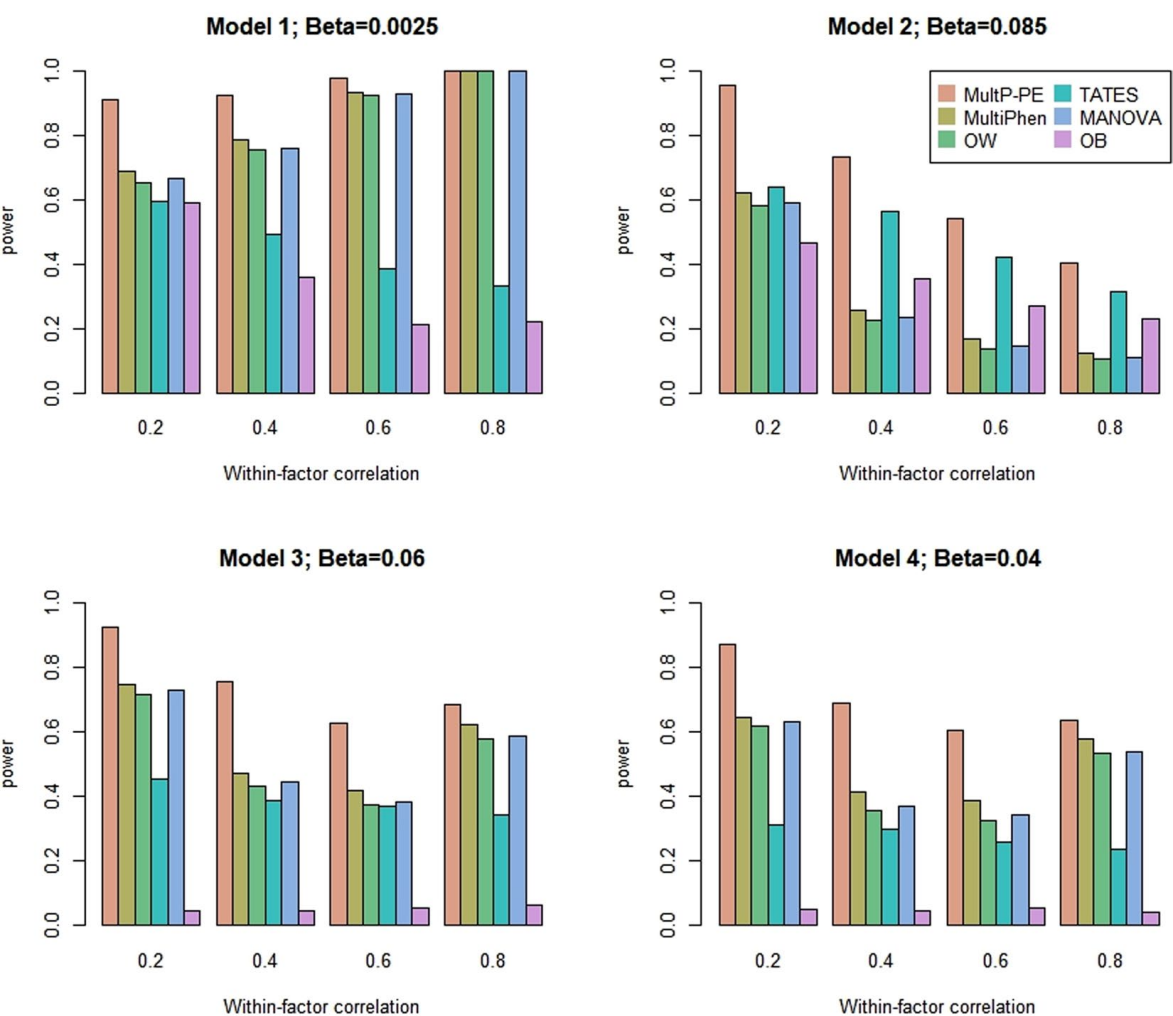
Power comparisons of the six methods as a function of within-factor correlation *c*^2^. The total number of phenotypes is *K* = 40, sample size is 1000, MAF is 0.3, and the between-factor correlation is 0.15. Significance is assessed at the 5% level.

Power comparisons of the six methods as a function of the within-factor correlation, *c*^2^, with *K* = 20 and 40 are given in Figs 3 and 4, respectively. As shown in these two figures: (1) the patterns of the power performance are similar to those in Figs 1 and 2; (2) when the within-factor correlation is increasing, the powers of all six tests have increasing trend or decreasing trend depending on different model settings. This pattern has been confirmed in Zhu’s paper^6^; (3) OB is the least powerful test except under model 2 with the within-factor correlation > 0.2.

Power comparisons of the six methods as a function of the between-factor correlation, *c*^2^*ρ*, with *K* = 20 and 40 are given in Figs S1 and S2, respectively. As shown in these two figures: (1) the patterns of the power performance are similar to those in Figs 1 and 2; (2) when the between-factor correlation is increasing, the powers of all six tests have increasing trend except for these under model 1; (3) MultP-PE is the most powerful test, while OB is the least powerful test except under model 2 with the between-factor correlation = 0.1.

In summary, MultP-PE is consistently the most powerful test among the tests we compared under all simulation scenarios.

## Real Data Analysis

Chronic obstructive pulmonary disease (COPD) is a terminology to describe progressive life-threatening lung diseases that causes breathlessness and serious illness, including emphysema, chronic bronchitis, refractory asthma, and some forms of bronchiectasis. A global prevalence of 251 million cases of COPD is reported in 2016 and it is estimated that COPD caused 3.17 million deaths in 2015^36^. The COPDGene aims to find inherited or genetic factors that associated with COPD. The COPDGene dataset includes 10,192 participants, 3,408 of them are African-Americans (AA), and 6,784 of them are Non-Hispanic Whites (NHW). Same as Liang *et al*.^37^, we considered Age, Sex, BMI, and Pack-Years as four covariates and selected seven quantitative COPD-related phenotypes (FEV1, Emphysema, Emphysema Distribution, Gas Trapping, Airway Wall Area, Exacerbation frequency, and Six-minute walk distance) in the following data analysis.

We deleted individuals and genotypes with missing data. After excluding missing data, a set of 5,430 NHW across 630,860 SNPs was used in the analysis. Then we adjusted the phenotypes for the covariates by applying a linear regression^14,17^. We regressed each phenotype on the four covariates, replaced original phenotypes with the residuals of the regression, and applied each of the six tests to detect the association between the covariates-adjusted phenotypes (residuals) and each SNP.

We used genome-wide significance level 5 × 10^−8^ to identify SNPs that are significantly associated with the seven COPD-related phenotypes. There were total 14 SNPs identified by at least one method (Table 2). All of the 14 SNPs had been reported to be associated with COPD by previous studies^38–50^. As shown in Table 2, MultiPhen identified 14 SNPs; OW, MANOVA, and MultP-PE identified 13 SNPs; TATES identified 9 SNPs; and OB did not identify any SNPs. The number of SNPs identified by MultP-PE was comparable to the largest number of SNPs identified by other tests and the COPD analysis results were consistent with our simulation results. We also performed individual phenotype analysis on each of the seven phenotypes. Table S1 gives the adjusted p-values (Bonferroni correction for multiple testing) to test each of the seven phenotypes on the 14 significant SNPs. We can see from Table S1, among the 14 SNPs, only nine SNPs are significantly associated with Emphysema Distribution at the genome-wide significance level. The number of SNPs identified by individual phenotype is the same as TATES and is less than the number of SNPs identified by four multiple phenotype analyses (OW, MANOVA, Multiphen, and MultP-PE), which showed that the simultaneous analysis of multiple phenotypes can increase power comparing with single phenotype analysis.

**Table 2:** Significant SNPs and the corresponding p-values in the analysis of COPDGene.

Chr	Position	Variant identifier	OB	TATES	OW	MANOVA	MultiPhen	MultP-PE
4	145431497	rs1512282	0.46	7.09 × 10^−13^	8.10 × 10^−14^	6.52 × 10^−14^	1.03 × 10^−9^	<1 × 10^−8^
4	145434744	rs1032297	0.49	6.22 × 10^−13^	1.11 × 10^−16^	1.11 × 10^−16^	7.69 × 10^−14^	<1 × 10^−8^
4	145474473	rs1489759	0.42	2.49 × 10^−16^	1.11 × 10^−16^	6.68 × 10^−17^	1.22 × 10^−16^	1.00 × 10^−8^
4	145485738	rs1980057	0.49	8.35 × 10^−17^	1.11 × 10^−16^	7.12 × 10^−17^	8.14 × 10^−17^	1.00 × 10^−8^
4	145485915	rs7655625	0.34	6.11 × 10^−9^	1.87 × 10^−9^	1.69 × 10^−9^	9.13 × 10^−17^	5.00 × 10^−8^
15	78882925	rs16969968	0.96	**5.40 × 10** ^**−8**^	2.05 × 10^−11^	1.77 × 10^−11^	7.84 × 10^−12^	<1 × 10^−8^
15	78894339	rs1051730	0.99	3.13 × 10^−8^	1.54 × 10^−11^	1.32 × 10^−11^	8.16 × 10^−12^	<1 × 10^−8^
15	78898723	rs12914385	0.99	2.76 × 10^−8^	1.64 × 10^−11^	1.41 × 10^−11^	1.48 × 10^−12^	<1 × 10^−8^
15	78911181	rs8040868	0.99	5.53 × 10^−10^	2.09 × 10^−12^	1.76 × 10^−12^	2.59 × 10^−12^	<1 × 10^−8^
15	78878541	rs951266	0.77	2.55 × 10^−9^	3.24 × 10^−12^	2.74 × 10^−12^	1.02 × 10^−11^	<1 × 10^−8^
15	78806023	rs8034191	0.87	**1.06** × **10**^**−7**^	2.42 × 10^−10^	2.14 × 10^−10^	7.74 × 10^−11^	<1 × 10^−8^
15	78851615	rs2036527	0.88	**1.62** × **10**^**−7**^	4.47 × 10^−10^	3.99 × 10^−10^	1.77 × 10^−10^	<1 × 10^−8^
15	78826180	rs931794	0.91	**1.23** × **10**^**−7**^	2.64 × 10^−10^	2.35 × 10^−10^	9.09 × 10^−11^	<1 × 10^−8^
15	78740964	rs2568494	0.27	**2.93** × **10**^**−5**^	**1.12** × **10**^**−7**^	**1.05** × **10**^**−7**^	4.23 × 10^−8^	**1.50** × **10**^**−7**^

The p-values of MultP-PE are evaluated using 10^8^ permutations. The p-values of OB, TATES, OW, MANOVA, and MultiPhen are evaluated using their asymptotic distributions. The bold out p-values indicate the p-values > 5 × 10^−8^.

## Discussion

For complex diseases in GWAS, the association between a genetic variant and each phenotype is usually weak. Analyzing multiple disease-related phenotypes could increase statistical power to identify the association between genetic variants and complex diseases. In this article, we developed a novel statistical method, MultP-PE, to test the association between a genetic variant and multiple phenotypes based on cross-validation prediction error. We showed that MultP-PE controls type I error rates very well and has consistently higher power than other methods we compared among all the simulation scenarios. Overall, MultP-PE is the most powerful test and has much higher power than the second most powerful test; OW, MANOVA, and MultiPhen have very similar performance; OB loses power dramatically when genetic effects have opposite directions on phenotypes; TATES is more powerful when the genetic effect only works on a portion of phenotypes. In real data analysis, MultP-PE identified 13 out of 14 significant SNPs, which is comparable to MultiPhen (14 out of 14).

## Supplementary information

Supplementary Information
