# Supplementary material for: Joint Analysis of Multiple Phenotypes in Association Studies based on Cross-Validation Prediction Error
Source: Sci Rep. 2019 Jan 31;9:1073. doi: 10.1038/s41598-018-37538-y (PMC6355816; doi:10.1038/s41598-018-37538-y)

*Supplementary Information*

**Joint Analysis of Multiple Phenotypes in Association Studies based on Cross-Validation Prediction Error**

Xinlan Yang^1^, Shuanglin Zhang^1^, Qiuying Sha^1^

^1^Department of Mathematical Sciences, Michigan Technological University, Houghton, Michigan, United States of America

**Correspondence**

Qiuying Sha, Department of Mathematical Sciences, Michigan Technological University, 1400 Townsend Drive, Houghton, MI 49931.

Email: qsha@mtu.edu

**Table S1.** Significant SNPs and the corresponding adjusted (Bonferroni correction for multiple testing) p-values for testing each of the seven phenotypes individually in the analysis of COPDGene.

| **Chr** | **Position** | **Variant identifier** | **Gas Trapping** | **Exacerbation Frequency** | **Emphysema** | **Airway Wall Area** | **Emphysema Distribution** | **Six-minute walk distance** | **FEV1** |
| --- | --- | --- | --- | --- | --- | --- | --- | --- | --- |
| 4 | 145431497 | rs1512282 | $2.81\times{10}^{-4}$ | $6.06\times{10}^{-3}$ | $1.64\times{10}^{-4}$ | 5.92 | $6.74\times{10}^{-9}$ | $6.12\times{10}^{-3}$ | $4.53\times{10}^{-3}$ |
| 4 | 145434744 | rs1032297 | $1.17\times{10}^{-5}$ | $7.33\times{10}^{-3}$ | $1.24\times{10}^{-5}$ | 5.26 | $7.64\times{10}^{-13}$ | $2.06\times{10}^{-3}$ | $2.99\times{10}^{-5}$ |
| 4 | 145474473 | rs1489759 | $1.51\times{10}^{-5}$ | $3.40\times{10}^{-2}$ | $8.82\times{10}^{-6}$ | 5.32 | 0 | $3.55\times{10}^{-4}$ | $3.96\times{10}^{-5}$ |
| 4 | 145485738 | rs1980057 | $1.92\times{10}^{-5}$ | $2.82\times{10}^{-2}$ | $1.82\times{10}^{-5}$ | 4.93 | 0 | $6.22\times{10}^{-4}$ | $7.18\times{10}^{-5}$ |
| 4 | 145485915 | rs7655625 | $1.45\times{10}^{-5}$ | $2.92\times{10}^{-2}$ | $1.29\times{10}^{-5}$ | 4.94 | 0 | $4.41\times{10}^{-4}$ | $4.13\times{10}^{-5}$ |
| 15 | 78882925 | rs16969968 | $4.88\times{10}^{-4}$ | 3.09 | $1.82\times{10}^{-7}$ | 5.13 | $3.13\times{10}^{-8}$ | 2.23 | $4.03\times{10}^{-6}$ |
| 15 | 78894339 | rs1051730 | $3.66\times{10}^{-4}$ | 3.28 | $1.82\times{10}^{-7}$ | 4.92 | $2.77\times{10}^{-8}$ | 2.15 | $3.54\times{10}^{-6}$ |
| 15 | 78898723 | rs12914385 | $4.77\times{10}^{-5}$ | 1.76 | $2.14\times{10}^{-7}$ | 1.91 | $5.32\times{10}^{-10}$ | $2.42\times{10}^{-1}$ | $1.49\times{10}^{-7}$ |
| 15 | 78911181 | rs8040868 | $1.07\times{10}^{-4}$ | 1.02 | $4.66\times{10}^{-7}$ | 2.55 | $2.43\times{10}^{-9}$ | $3.37\times{10}^{-1}$ | $8.23\times{10}^{-8}$ |
| 15 | 78878541 | rs951266 | $5.86\times{10}^{-4}$ | 3.19 | $1.67\times{10}^{-7}$ | 5.38 | $5.42\times{10}^{-8}$ | 2.77 | $6.39\times{10}^{-6}$ |
| 15 | 78806023 | rs8034191 | $2.45\times{10}^{-3}$ | 3.06 | $1.61\times{10}^{-6}$ | 4.40 | $1.03\times{10}^{-7}$ | 3.21 | $2.80\times{10}^{-5}$ |
| 15 | 78851615 | rs2036527 | $3.35\times{10}^{-3}$ | 3.49 | $3.00\times{10}^{-6}$ | 4.45 | $1.64\times{10}^{-7}$ | 2.14 | $2.09\times{10}^{-5}$ |
| 15 | 78826180 | rs931794 | $1.39\times{10}^{-2}$ | 3.70 | $4.84\times{10}^{-6}$ | 3.76 | $1.23\times{10}^{-7}$ | 2.38 | $5.94\times{10}^{-5}$ |
| 15 | 78740964 | rs2568494 | $3.40\times{10}^{-2}$ | 6.46 | $3.08\times{10}^{-5}$ | 6.79 | $2.01\times{10}^{-4}$ | 1.67 | $1.97\times{10}^{-4}$ |

**Figure S1**. Power comparisons of the six methods as a function of between-factor correlation $c^{2}\rho$. The total number of phenotypes is $K=20$, sample size is 1000, MAF is 0.3, and the within-factor correlation is $c^{2}=0.25$. Significance is assessed at the 5% level.


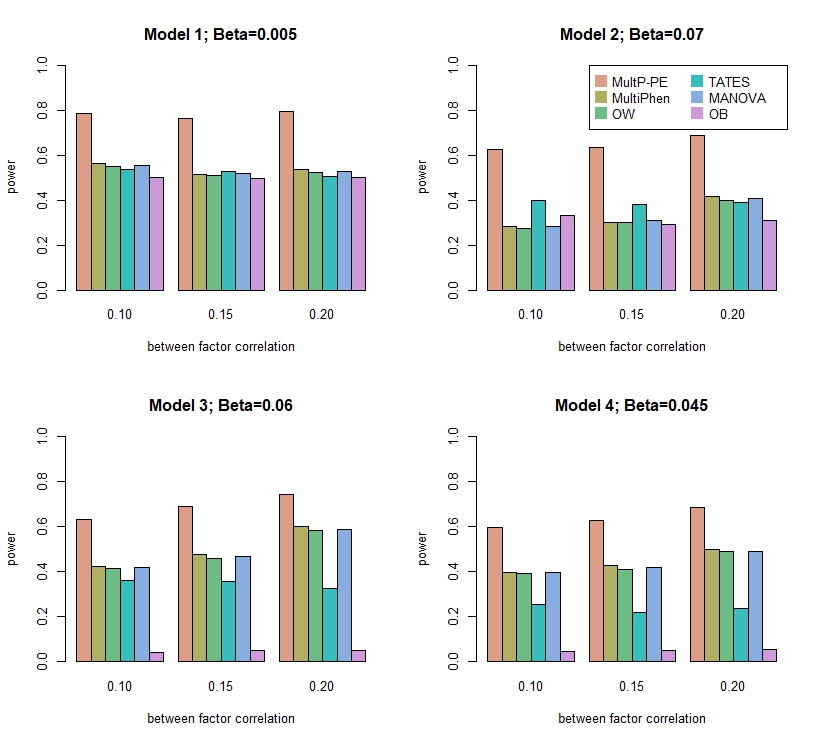


**Figure S2**. Power comparisons of the six methods as a function of between-factor correlation $c^{2}\rho$. The total number of phenotypes is $K=40$, sample size is 1000, MAF is 0.3, and the within-factor correlation is $c^{2}=0.25$. Significance is assessed at the 5% level.


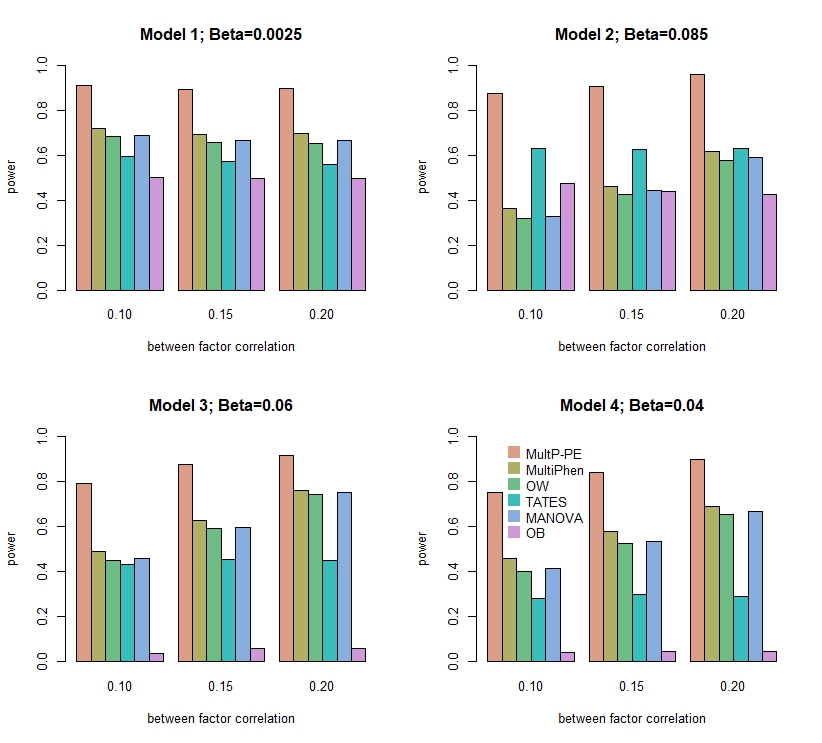

Supplement: Supplementary file 1 — Supplementary Information [file 41598_2018_37538_MOESM1_ESM.docx]
